# Global health, planetary health, One Health: conceptual and ethical challenges and concerns

**DOI:** 10.1007/s11017-024-09670-6

**Published:** 2024-05-25

**Authors:** Eduardo Missoni

**Affiliations:** SDA Bocconi—Management School, Milan, Italy

**Keywords:** Global health, Planetary health, One Health, Definitions, Ethical challenges

## Abstract

The Covid-19 pandemic has dramatically shown the level of interconnectedness of the human population, the direct relation between human health and the ecosystem, as well as the enormous ethical challenges required for a global response. Relatedly, society has been directly confronted by issues of ‘Global health,’ both in terms of awareness of health conditions and health systems resiliency all around the world, as well as in terms of governance of the worldwide response and its implications at national and local levels. While Global health is often used as a cosmetic label for neocolonial approaches, it is really an interdisciplinary approach consisting of the interaction between globalization and the determinants of health. Thus, it involves the ecosystem and its transformation and implies a systemic ‘One Health’ decolonized approach in the definition of its strategies. The Covid-19 pandemic has highlighted the inequities and the limits of the current hegemonic Global health system governance; calling for ethics to provide a renewed, comprehensive, inclusive, and decolonized conceptualization of Global health.

## Introduction

The Covid-19 pandemic has dramatically shown the level of interconnectedness of the human population; the direct relation between human health, its societal determinants, and the ecosystem; as well as the enormous ethical challenges required for a global response in terms of equity, transparency of information, democracy, freedom of expression, human rights, use of public resources, and conflict of interests—among others—that go beyond individually focused ethics [1].

The pandemic has also confronted society with ‘Global health’ (GH), both in terms of awareness of health conditions and health systems resiliency, and in terms of governance of the worldwide response and its implications at national and local levels.

GH, understood as ‘Health and well-being, for all at all ages,’ as established in the Sustainable Development Goal no. 3 of the Agenda 2030, is a socially desirable objective. However, reluctancy—even (or especially) among medical professions—to recognize the role of societal determinants of health has resulted in a top-down, biomedical, technological, and often dogmatic approach to health. By missing the fundamental point that health is “not merely the absence of disease or infirmity” [2], it closes doors to any alternative cosmovision and practice.

In this way, “the self-interested silence of the knowledgeable, the opportunism of the powerful (no less than WHO), the connivance of politicians” [3, p. 581] have contributed to the decades-long disregard of the vision and awareness of the ‘common planetary destiny’ that all humans share among themselves and with all living species on Earth.

Despite the frequent reference to GH in the narrative surrounding the pandemic, a univocal understanding of GH study and practice is still missing; hence, the term has been widely used and misused. Thus, it is necessary to clarify the concept and to understand its origins, meaning, use and, for the purposes of this essay, its ethical implications.

In the mid 1990s, international studies progressively shifted focus away from the nation-state as a fundamental analytical unit and inter-national relations, to a more comprehensive emphasis on issues related to the acceleration of globalization: increasing interconnectedness and interdependence, the emergence of new transnational forces, global power structures and processes, the consequent transformation of global society, and the impact of those processes on the ecosystem.

Indeed, some engaged in GH as a new field of study, research, and practice that investigates the interaction between the multidimensional globalization process and health. They adopted an ethical approach focused on equity, human rights, and health determinants, with a clear understanding of the ecosystemic and planetary dimensions of the issues at stake. To them, the attainment of GH as a goal—on the World Health Organization agenda since 1977 as “Health for all by the year 2000” [4]—would require global responses to the above issues rooted in local awareness and action, along with moral responsibility toward future generations [3].

However, the reference to the global dimension of phenomena has become uncritically common and fashionable. Thus, the health field has suffered the tendency to re-label as “global” various issues and modalities of the past, “which, in turn, has led to conceptual and empirical imprecision” [5, p. 137]. In other words, almost everything has been thrown into the ‘global’ pot, with ‘global’ being used to label issues and modalities previously defined by terms (such as international) that have proven perfectly adequate. In doing so, many have lost sight of what originally motivated the shift from ‘international’ to ‘global,’ i.e. the ongoing transformation of global society.

Accordingly, the purpose of this paper is twofold. First, it conceptually clarifies the comprehensive nature of GH to emphasize the connection between human health and the health of the ecosystem. Accordingly, GH will be discussed alongside the concepts of Planetary Health (PH) and One Health (OH) in response to the prevailing reductionist and bio-medical interpretations of GH. Second, this paper will highlight the neocolonial characteristics and ethical implications of GH policies and of emerging self-defined GH organizations implementing them. To do this, the origins and meanings of GH, PH and OH are first presented. Then, the inequities and the limits of the current hegemonic GH system and its governance—as revealed in the pandemic—are exposed, calling for ethics to provide a renewed, comprehensive, inclusive, and decolonized approach to GH.

## Global health

Over the last two decades, international and bilateral institutions, as well as private organizations and the media, have increasingly referred to initiatives in health beyond national borders as GH initiatives or programs. GH was thus appropriated as a fast-growing market for transnational corporations, and new transnational public–private/multistakeholder partnerships were born under the global flag.

Accordingly, the offer of collegiate courses in GH has boomed over the last two decades or so. New journals have been dedicated to this field of study. The number of articles using GH in the title or abstract in MEDLINE increased by nearly 30-fold between 2001 and 2019 [6]. In 2009, Richard Horton, chief editor of *The Lancet*, expressed that GH was becoming a critical aspect of the educational, scientific, and moral mission of universities [7]. However, there was widespread discrepancy about what GH would stand for (including its ‘moral mission’) and about the content of GH initiatives, research, and courses offered around the world.

Primarily defined by institutions in the Global North, GH continued to be expressed in terms of the Global North’s relations with low-income countries. For example, here is a definition of GH reportedly given by the United States’ National Institutes of Health: “Research, teaching, clinical care, prevention, and outreach activities directed towards addressing health concerns that contribute a significant burden of disease and disability in low- and middle-income countries and that are of general concern to the international health community” [8, p. 386] (i.e., initiatives in the context of—and in continuity with—the traditional neo-colonial North–South ‘development aid’ relation). Indeed, for low-middle income ‘aid’ recipient countries, GH was business as usual: “An activity developed through the lens of rich countries, ostensibly for the benefit of poor countries, but without the key ingredients of a mutually agreed, collaborative endeavor” [8, p. 384].

Such an approach to GH, still largely dominant, has been criticized as “the newest depoliticized and de-historicized iteration of colonial medicine” which is “often taught uncritically, without a deeper reflection on Western scientists’ social position as part of the Global North’s scientific enterprise and related positionality; power dynamics; historical context; and contemporary colonial approaches such as top-down GH governance and programming” [9]. Nevertheless, in the face of the phenomenon of neoliberal capitalist economic globalization, autonomous analyses have been developed that differ from the predominantly North-centric approaches to GH [10, 11].

In many cases, the term GH has been used to refurbish pre-existing courses and initiatives in international health, tropical medicine, and others in a mere response to marketing needs [12]. This is really just “a cosmetic re-labeling of old patterns, objects, and interests” with no impact on social innovation and with little attempt to fully explain the definition and contents of the GH terminology [13, p. 19].

There is also deep discordance about the characteristics of GH policies and projects and how they are implemented. The impact of neoliberal globalization on population health and health systems highlights “the macro institutional and place-specific factors that intersect within GH spaces to produce this discordance” [14, p. 60]. Most of the health issues and policies addressed by so-called GH advocates remain local and encompass diverse and often contradictory objectives.

The WHO and other multilateral organizations have been overwhelmed by philanthropic capitalism and market interests. Driven by business models aimed at revenue generation and dependent on knowledge control and the promotion of exclusive technology-based systems, this approach to GH heightens inequalities and lacks accountability to citizens [15].

Additional ethical concerns derive from the securitization of GH resulting from pandemic threats (e.g., SARS, H5N1 influenza, H1N1 influenza, Zika, Ebola, and now Covid-19) over the last two decades. This reframing of health problems as issues of security and territory often leads to authoritarian measures and the labeling of ‘contaminated’ individuals as dangerous threats [14]. ‘We are at war’ with the virus was a common narrative during Covid-19 in response the challenge posed by the virus [16], appropriating an ancient metaphor used to introduce exceptional emergency responses, to criminalize dissent, and to increase police powers and surveillance [1].

GH as an updated reprint of previous concepts tends to marginalize the perspective of social medicine and undermine the social determinants of health. For example, biomedical reductionism has narrowed the GH agenda to focus on singular diseases, emphasizing technological and pharmaceutical solutions while avoiding any exploring and understanding of the political, social, cultural, economic, and environmental determinants of health. However, life cannot be reduced merely to its biological expression. Referring specifically to the Covid-19 pandemic, Horton states that it is imperative to resist the “biologicalization” of this disease and instead insist on a social and political critique that leads to framing disease as a pathology of society [17]. In other words, it is imperative to identify and fight against the causes of the causes: the dominant neoliberal and market-oriented societal model which threatens the sustainable development goals of the global Agenda 2030 [18]. Moreover, reflecting on the recent pandemic, ten Have introduces the notion of a “common home” to articulate the shared—rather than individualistic—dimension of the pandemic experience, and extends the reflection to other global threats [1].

Thus, “what should be taught when global health is taught?” [19]. And, by extension, what is the scope of research and practice in GH? What are the goals and scope of governance in GH? In a word, what is the domain of GH?

Bozorgmehr identified four ways in which the term “global” could be understood in the health literature: (1) “worldwide” or “everywhere”; (2) health issues that are not limited by national boundaries (e.g. pandemics and the spread of infectious disease); (3) a broad-spectrum approach that is multidisciplinary in character; and (4) supra-territoriality that allows GH to focus “on the globality of the social determinants of health and the power relations in global social space” [13, p.19].

While helpful, the four interpretations that Bozorgmehr proposes for the term ‘global’ miss two additional aspects. The first (and possibly the most important) is related to the Globe, the Planet, Mother earth (*Pacha mama*, in the expression of many Andean populations), the entire ecosystem. The second imagines ‘global’ as considering or including all parts of something (i.e., an integral, comprehensive, inclusive, and systemic view). From such a perspective, Covid-19 is a “syndemic,” i.e. an event characterized by biological and social interactions, which cannot be understood and adequately faced without “a vision encompassing education, employment, housing, food, and environment” [20, p. 874] and ethics directing action at all levels.

Rieder synthesizes GH as “a set of processes that occur at the intersections of transnational networks” [14, p. 60], i.e., beyond the interactions between nation-states. This work reveals the inadequacy of a GH concept limited to “international” public health [19].

Furthermore, the broad-spectrum approach with the inclusion of multiple consolidated disciplines (biology, health sciences, sociology, political sciences, diplomacy, economics, anthropology, environmental sciences, and others) extends the understanding of GH beyond just a transnational dimension of public health [21]. For the same reason, GH cannot be identified as a self-standing discipline. It is more correct to define it as an “area” of studies and practices at the crossroads of many disciplines with a complexity that—in the typically compartmentalized structure of modern studies—may have limited the education of practitioners and the emergence of an intellectually robust field [22].

Whatever the nuances of the definition, it must be emphasized that engaging with the promotion of health in a global dimension implies an ethical intergenerational commitment and an overarching ideal: equity. As it was pointed out by the Commission on the Social Determinants of Health, addressing the current inequities in worldwide health status has become one of the primary goals in all GH studies [23].

Possibly the most cited definition is the one coined by Koplan in 2009:

an area for study, research, and practice that places a priority on improving health and achieving health equity for all people worldwide. Global health emphasizes transnational health issues, determinants, and solutions; involves many disciplines within and beyond the health sciences and promotes inter-disciplinary collaboration; and is a synthesis of population-based prevention with individual-level clinical care [12, p. 1995].

In this definition, as in many other propositions, a specific link is missing regarding the acceleration of the globalization process “which is a force in shaping the health of populations around the world” [12, p. 1994] and, as shown above, is at the origins of and is a defining element of GH as a field of study. Instead, the link “to individual-level clinical care” is misleading and possibly introduced by those authors who support ‘GH training’ by clinical rotation in low-income countries to provide medical students with “broader knowledge of tropical disease and newly emerging infections” [24, p. 226]. These kinds of experiences are obviously great opportunities for intercultural exposure for medical students but should not be confused with studies in GH which aim at disentangling the societal powers and processes determining the policies and health of global populations. Rather, ‘GH training’ should emphasize “interactions with national and local systems” [25, p. 3], where the ‘systems’ are by no means limited to a particular health system, but involve the ecosystem as well as political, economic, social, cultural systems. In other words, it ought to emphasize a systemic approach with health at its center.

Swiss scholars have also put forward a definition for academic GH involving education and research that reaffirm the systemic and ecologic, transdisciplinary approach, but with an added reference to “the normative framework of human rights” and “innovative, integrated and sustainable” solutions [26]; the latter an attribute evocative of the Global sustainable development agenda 2030 launched in 2015.

## Planetary health

More recently, the concept of “planetary health” (PH) was launched by *The Lancet* and supported by the Rockefeller Foundation. Its scope supposedly goes beyond the boundaries of “the existing GH framework to take into consideration the natural systems upon which human health depends” [27, p. 847]. However, as explained above, in its full meaning, GH already includes “the natural systems;” it is the dominance of a reductionist, bio-medical, and neo-colonialist understanding of GH that has suggested the need for a new branch of health sciences. Curiously, the main supporter of PH presented it as “A New *Discipline* in Global Health” [28], implicitly recognizing it as part of the interdisciplinary area of studies above described as GH.

In the original “Manifesto,” PH is presented as “an attitude towards life and a philosophy for living” [27, p. 847]. Its emphasis on people and equity, the recognition of the impact of neoliberal globalization on health and sustainability of human development, as well as the focus on “interdependence and the interconnectedness of the risks we face” [27, p. 847], all belong to a comprehensive understanding of GH. The ecosystem is undoubtedly one of the most important determinants of human health and, in that sense, the PH’s emphasis on linking human health and a healthy planet is greatly welcome.

## One Health

Another holistic approach that examines human health in relation to life on the planet—with the aim of improving or safeguarding animal and human health—is the perspective of One Health (OH).

The OH approach has been traced back to ancient times but is increasingly used. It emphasizes that human health and animal health are interdependent and bound to the health of the ecosystems in which they live. OH also takes a collaborative, multisectoral, and trans-disciplinary approach—to be developed from the local to global level—to achieve optimal health and well-being outcomes [29]. Accordingly, OH incorporates a whole-of-society approach to policy making [30].

Yet, a univocal definition of OH is lacking. Indeed, some consider OH too wide a concept encompassing individual, population, and ecosystems health, and other concepts such as ‘Eco-health’ (focused on the relationships between human and animal health and the environment); ‘One medicine’ (used in relation to transmissible or contagious zoonotic diseases); ‘Comparative medicine’ (based on the idea of comparing humans to one or more chosen animal species); ‘Translational medicine’ (used to illustrate how different knowledge in basic scientific disciplines can be ‘translated’ into new or improved therapies, procedures, diagnostic tools, or policies for individuals and populations); ‘Zoobiquity’ (aimed at integrating human and veterinary medicine and biology into an interdisciplinary approach); and ‘Evolutionary medicine’ (adopting biological ideas of macro- and micro- evolution, fitness and environment to medical thinking) [31].

On this basis, it is evident that the concept of OH perfectly fits within a comprehensive understanding of GH, especially when exploring issues that extend beyond national boundaries and involve transnational processes and actors.

## Conclusions

GH is the answer to the need for “a ‘planetary’ vision for One Health,” encompassing both the OH and PH frameworks “that facilitate going from ‘local to global’, or more accurately ‘molecular to planetary’, to address the health, well-being and sustainability of humans, animals and the environment” [32].

Undoubtedly, to be effective in the policy arena, disparate groups of scholars and their flagship approaches (OH, PH, and others) must be kept together [30]. In that sense, GH—well understood in its comprehensive meaning—perfectly represents the needed umbrella definition. GH allows space for the whole of society/whole of Planet action, involving multiple disciplines beyond just the health sciences.

However, whatever definition of GH may be adopted, it must be understood that research, education, practice, governance and policy-making in this domain have been suffering from a neocolonial approach with multiple forms of global power—in which transnational corporations, global philanthropy and, increasingly, multistakeholder initiatives and marginalized multilateral institutions—combine to perpetuate colonial structures of low-income countries’ dependency on the Global North. In this sense, awareness is emerging and nurturing an intense debate on the imperative need to ‘decolonize global health’ as the global Covid-19 pandemic response also revealed with stark clarity [9, 33–35].

In the process of decolonizing GH, a counter-hegemonic vision of GH must be adopted, such as the one proposed from a Latin American perspective [10, 11]. In crafting a ‘Global Southern’ view and practice of GH, the linking of local to global becomes essential and compels one to focus on the local conditions and sociocultural foundations of health and illness; thereby “bringing local history center stage and drawing extensively on the social sciences” and tracing the geopolitical imbalances that are at the core of GH issues [36]. Widening one’s view and practice from the global South—with the emphasis on collaborative approaches—the conceptualization of GH should be left open to different cultures to let it evolve along with GH research, teaching, policy, and practice [37].

In sum, the Covid-19 pandemic has exacerbated inequities, highlighted the limits of the current hegemonic GH system, and exposed unethical influences in its governance. It has made social and communal relations problematic, hence the need for a global ethical perspective [1]. Ethics must provide a renewed conceptualization of GH, one that challenges avoidable structural inequalities that exacerbate the unjust, iniquitous character of today’s global society.
